# Knocking Out AMR project review

**DOI:** 10.1099/jmm.0.001900

**Published:** 2024-09-10

**Authors:** Lovleen Tina Joshi, Catrin E. Moore

**Affiliations:** 1 Peninsula Dental School, Faculty of Health, University of Plymouth, Plymouth, UK; 2 City St George’s, University of London, London, UK

**Keywords:** Antimicrobial Resistance

Antimicrobial resistance (AMR) is a global health concern estimated to have killed between 1.7 and 4.95 million people in 2019, with an annual cost of $900 billion globally [1]. The immediate concern is the looming post-antibiotic era as we have a lack of new antibiotics coming through the pipeline putting people at risk of untreatable infections after routine surgeries. In many places we simply do not know how bad the situation is, and we are unable to determine the cause of infection and the correct treatment because of the lack of available diagnostic tests or if available their prohibitive cost. If we do not have the evidence to treat patients in clinics, a huge gap exists in the evidence needed to inform a public health response. If available, this evidence would inform preventative measures or effective interventions to minimize the catastrophe that is AMR. The breadth, scale and complexity of the AMR problem demands the breaking down of historical silos and an urgent, global commitment to collaborative working across sectors and borders. With this backdrop, together with the urgency and a heightened interest from policy-makers with the updating of National Action Plans (NAPs) for AMR [2] and the United Nations High Level Meeting discussing AMR in 2024, the Microbiology Society launched the Knocking Out AMR project in October 2023.

Solutions to the AMR crisis are desperately needed and microbiologists are at the forefront of developing innovative approaches to decreasing the devastating effects of AMR. The Knocking Out AMR project is a 4 year, ambitious, forward-thinking scheme of work aiming to promote effective solutions to minimize AMR, through cross-disciplinary and multi-sector collaborations globally within the One Health [3,4] context. The project is concentrating on areas where the Microbiology Society and its members could effectively stem the rising tide of AMR. The project focuses on solutions for surveillance, diagnostics, vaccines, novel antimicrobials and policy to improve the awareness and understanding of the urgency of the AMR crisis, supporting action for solutions to minimize AMR. As co-leads of the project, we convened an oversight group, supported by the impact and influence committee within the Microbiology Society, to ensure accountability of the project.

The Knocking Out AMR project hosted a series of solution-oriented, invite-only workshops with 135 experts from a range of sectors, including academia, industry, clinical and veterinary settings, regulatory bodies, funding bodies, national and international policy agencies, not-for-profit organizations and knowledge exchange networks. The experts covered the three One Health settings (humans, animals and the environment) and a wide range of micro-organisms (bacteria, fungi, viruses and parasites). They were invited to improve our understanding of current systems and the way they prevent the development and implementation of solutions to minimize AMR. Society members were also invited to feed into an online survey with 400 members joining, which has provided feedback on the new UK AMR NAP. The workshops focused on four solution areas, namely diagnostics, surveillance, therapeutics and vaccines, and were hybrid in nature with both in-person and online attendance.

The reports from these meetings, focusing on each solution area in turn, are published on our website today, and we are grateful to all of the note takers at each meeting. The workshop used the complex system approach [5,6] to visualize a world in which one of the four subjects (diagnostics, surveillance, therapeutics or vaccines) were being used effectively to minimize AMR, then to describe the barriers preventing this vision and the interventions necessary to enable the vision. The interactions between the barriers, intervention and the vision were all discussed at the workshops in small groups and in one group to ensure all opinions were heard. Each of these workshops are described in detail on the website, with an overall combined systems map produced for each of the four key topics. The visions for each topic included some overlapping themes, such as universal access, low-cost, proactive surveillance, comparable data that are shared widely leading to better and more usable evidence, breaking down silos and increasing collaboration, an understanding of the key drivers of resistance, optimized use of antimicrobials, a functioning economic market for all four areas, and availability and affordable access for all across the One Health paradigm.

The Knocking Out AMR project has high aims and ambitions for an impactful plan of work, and we are building our engagement with experts, society members and policy-makers to ensure our vision represents our stakeholders and is clear. We are hosting a new online series called AMR in Focus with online talks beginning in September; the first focuses on engagement with policy-makers (our website), and following talks will alternate between science and professional development. We are also building resources for education, engaging with health agencies to support ongoing AMR NAP activities across the UK and supporting our Microbiology Society Champions to plan World Antimicrobial Awareness Week activities in November 2024. In line with our outreach, we are linking Microbiology Society experts on AMR with media contacts. The Society is working with external societies and influential groups to support future solutions in our priority areas and there will be a dedicated space for AMR work at the Microbiology Society annual conference. The last 12 months have been a whirlwind of activities, and the coming year holds much promise and hope with the upcoming political meetings focusing on AMR.

## Abbreviations

AMR: antimicrobial resistance
NAP: National Action Plan
